# Associations of Serum Albumin With Disability in Activities of Daily Living, Mobility and Objective Physical Functioning Regardless of Vitamin D: Cross-Sectional Findings From the Chinese Longitudinal Healthy Longevity Survey

**DOI:** 10.3389/fnut.2022.809499

**Published:** 2022-02-24

**Authors:** Xueqin Li, Xingqi Cao, Zhimin Ying, Jingyun Zhang, Xiaoyi Sun, Emiel O. Hoogendijk, Zuyun Liu

**Affiliations:** ^1^Center for Clinical Big Data and Analytics of the Second Affiliated Hospital and Department of Big Data in Health Science School of Public Health, Zhejiang University School of Medicine, Hangzhou, China; ^2^Department of Orthopedic Surgery, The Second Affiliated Hospital, Zhejiang University School of Medicine, Hangzhou, China; ^3^Department of Epidemiology and Data Science, Amsterdam Public Health Research Institute, Amsterdam UMC-location VU University Medical Center, Amsterdam, Netherlands; ^4^Alibaba-Zhejiang University Joint Research Center of Future Digital Healthcare, Hangzhou, China

**Keywords:** serum albumin, activities of daily living, mobility, objective physical functioning, disability, older adult

## Abstract

**Objective:**

To examine the associations of serum albumin, a nutrition indicator, with disability in activities of daily living (ADL), mobility, and objective physical functioning among Chinese older adults.

**Materials and Methods:**

Cross-sectional data of 2233 older adults (≥65 years) who participated in the 2011/2012 main survey of the Chinese Longitudinal Healthy Longevity Survey (CLHLS) and the 2012 biomarker sub-study was used. Serum albumin was measured by immunoturbidimetric assay. Physical functioning included subjectively (ADL and mobility) and objectively measured disability (standing up from a chair, picking up a book from the floor, and turning around 360°). Multivariable logistic regression models were performed.

**Results:**

After adjusting for age and sex, compared with participants in the lowest quartile group of serum albumin, those in the highest quartile group had 45% lower odds of disability in ADL (odds ratio [OR]: 0.55; 95% confidence interval [CI]: 0.38, 0.80); 48% lower odds of disability in mobility (OR: 0.52; 95% CI: 0.38, 0.71); 46% lower odds of disability in standing up from a chair (OR: 0.54; 95% CI: 0.34, 0.85); and 37% lower odds of disability in picking up a book from the floor (OR: 0.63; 95% CI: 0.40, 0.97). We did not observe a statistically significant interaction effect between serum albumin and vitamin D on disability in physical functioning.

**Conclusion:**

Serum albumin level was associated with physical functioning among Chinese older adults, regardless of vitamin D level. The findings indicate that appropriate management of poor nutritional status, in particular low serum albumin levels, may contribute to maintaining physical functioning in older adults.

## Introduction

By 2050, China will have 400 million adults aged 65+ years (1). Among this older population, the prevalence of disability increases rapidly with age, posing huge burdens to the family and healthcare systems (2). Having difficulty or dependency in performing activities essential for self-care and independent living in daily life among older adults, which is termed as physical disability (3, 4). Physical disability may include basic activities of daily living (ADL) and mobility, which emphasizes different aspects of functioning. The causes of physical disability with aging are likely to be multifactorial, making disability management to be complicated. Biomarkers, usually measured in blood, urine, or other biospecimens, serve as important indicators of physiological states or conditions. Identifying biomarkers of physical disability could provide not only clues for early screening and preventive interventions but also insights into the underlying mechanisms (5–7).

Albumin, the most abundant protein in the blood, is a significant indicator of malnutrition. Recently, lower serum albumin level has been linked to a decline in physical functioning among older adults in various studies (8–13). However, these studies were limited by a lack of comprehensive assessment of physical functioning, e.g., they did not combine objective and subjective measures of physical functioning (8, 10, 12, 13), or targeting populations without representativeness (9, 11, 13). As aging-related physiological changes are linked to the function of the whole body, a relatively multi-dimensional approach to physical functioning is crucial. In addition, recent studies have revealed that a low level of vitamin D is also significantly associated with the decline in physical functioning among older adults (14–17). Malnutrition and vitamin D deficiency are very common in older adults (18, 19), and are correlated as well. As a major secondary carrier of vitamin D (20), changes in serum albumin levels are likely to cause changes in serum vitamin D levels (21). Thus, it is likely that serum albumin and vitamin D synergistically play a role in physical functioning, which, however, has been less investigated in previous studies (22).

In the context of rapid population aging in China, this study aimed to examine the associations of serum albumin with disability in ADL, mobility, and objective physical functioning among Chinese older adults. Furthermore, we aimed to evaluate the interaction between serum albumin and vitamin D on physical functioning in the same population. We used data from the 2011/2012 main survey and the 2012 biomarker sub-study of the Chinese Longitudinal Healthy Longevity Survey (CLHLS), a national survey in China.

## Methods

### Study Population

CLHLS was established in 1998 as a national survey and was designed to investigate determinants of healthy longevity in Chinese older adults. Details of the methodology and the study design have been previously reported (23). In short, the CLHLS was conducted in a random sampling of 50% of cities and countries in 23 provinces across China, accounting for 85% of the total population. The CLHLS tried to interview all centenarians who agreed to participate in the study. To ensure representativeness, the CLHLS conducted a target random sampling design and performed identical interviews with approximately equal numbers of young-older adults (aged 65–79 years), octogenarians, and nonagenarians who lived in the same area as the centenarians. To fill the gap that some objective medical aspects such as biomarkers are not available in CLHLS, the researchers have added in-depth surveys in seven longevity regions where the number of centenarians is large as part of the 5th wave of CLHLS in 2009, and eight longevity regions (plus a new one based on the survey in 2009) as part of the 6th wave of CLHLS in 2012. The in-depth surveys included more comprehensive health examinations by medical personnel and collected urine and blood samples during home interviews. This in-depth surveys were called the biomarker sub-study of CLHLS, or Healthy Aging and Biomarkers Cohort Study (HABCS) (2, 24). The Duke University and the Research Ethics Committees of Peking University granted approval for the Protection of Human Subjects for the CLHLS and all participants provided informed consent.

**Figure 1:**
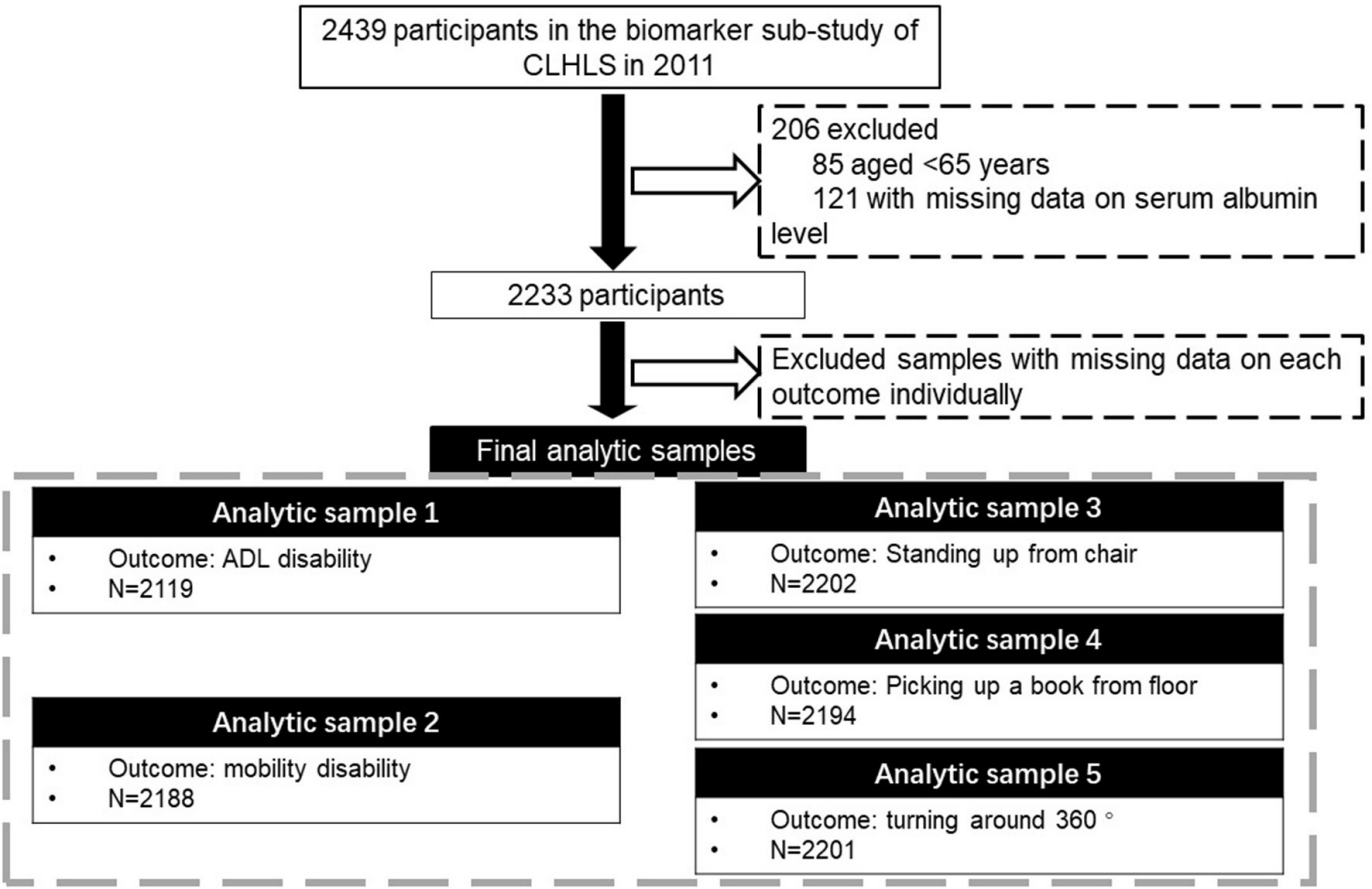
Flowchart of assembling study participants. CLHLS, Chinese Longitudinal Healthy Longevity Survey; ADL, activities of daily living.

In this study, we included participants from the 2012 biomarker sub-study and the 2011/2012 main survey of CLHLS (*N* = 2439). We excluded participants aged below 65 years (*N* = 85) or with missing data on serum albumin levels (*N* = 121), and had a sample of 2233 participants, with no missing data on covariates. Further, due to the varying numbers of missing data on each outcome, different analytic samples for each outcome were assembled (Figure 1).

### Assessment of Serum Albumin Levels and Serum Vitamin D Levels

About 5 ml fasting venous blood samples were collected from participants by trained medical staff with heparin anticoagulant vacuum tubes. All blood samples were centrifuged at 2,500 rpm at 20°C for 10 min. The separated specimens were frozen at −20°C and transferred to Central Laboratory in Beijing, where they were stored at −80°C until biochemical analysis. Serum albumin level and vitamin D levels were measured by immunoturbidimetric assay and enzyme-linked immunosorbent assay (Immunodiagnostic Systems Limited, Bolton, UK), respectively. Further details are provided elsewhere (25, 26).

Serum albumin levels were divided into quartiles and the cutoff values were 36.8, 40.1, and 43.3 g/L. In addition, we divided serum albumin and vitamin D into a dichotomous variable. The cutoff value of serum albumin was 35 g/L, recommended to define hypoalbuminemia in literature (27). Similarly, the cutoff value of serum vitamin D was 50 nmol/L, recommended to define vitamin D deficiency (28).

### Assessment of ADL Disability and Mobility Disability

In the CLHLS survey, six items of daily self-care ability were collected from each participant based on the Katz index: dressing, bathing, transferring, toileting, continence, and eating (29). Each item was asked with three answers, i.e., “complete independence,” “partially dependence,” and “complete dependence” (30). As done in previous studies, ADL disability was defined as present if participants needed any assistance (i.e., “partially dependence,” and “complete dependence”) in performing at least one of the six self-care activities (31).

The status of mobility function was assessed by asking participants the question: “Can you walk continuously for 1 kilometer at a time by yourself?” As done in previous studies, mobility disability was defined as present if participants reflected any difficulty in this task (32).

### Assessment of Disability in Objective Physical Functioning

Objective physical functioning was measured through three tasks (protected by trained technicians) in every participant's home. These tasks included picking up a book from the floor, standing up from a chair, and turning around 360°. Thus, three dichotomous indicators were used to represent the presence or absence of disability in objective physical functioning. If participants were not able to perform one task independently, they were defined as having a disability in this task (33).

### Covariates

According to previous studies, we found that some population characteristics (e.g., age, sex, ethnicity, and so on) (34–39) and lifestyles (e.g., currently smoking, current consuming alcohol, and regular exercise) (40–43) may confound the associations between serum albumin and physical functioning. Thus, as done in the previous study based on CLHLS (44), the following covariates were included in our analysis: age, sex, residence (rural vs. other), ethnicity (Han vs. other), education (≥1 year of education vs. no education), economic independence (yes vs. no), lifetime primary occupation (white-collar occupation vs. others), and being in receipt of adequate medications (yes vs. no); co-residence with children (yes vs. no) and current marital status (married vs. others) were used to assess participants' social support and contact; lifestyle consisted of consuming alcohol (yes vs. no), currently smoking (yes vs. no), and regular exercise (yes vs. no). In addition, chronic diseases were included in our models for both serum albumin and physical functioning could be influenced by major chronic diseases. Five self-reported chronic diseases (diabetes mellitus, hypertension, heart disease, stroke/cardiovascular disease (CVD), and respiratory diseases) diagnosed by doctors were included. Furthermore, we additionally added mild cognitive impairment (MCI) (defined by Mini-Mental State Examination (MMSE) score below 24) in our analyses, for it may influence physical functioning (45). Body mass index (BMI) was also included. BMI was calculated as weight divided by height squared (kg/m^2^). The height (in centimeter) and body weight (in kilogram) of participants were measured by interviewers during the survey (46).

### Statistical Analyses

Basic characteristics of study participants were summarized in the total sample and by serum albumin quantile groups. Continuous variables were summarized by mean ± standard deviation (SD) and categorical variables were summarized by numbers and percentage. The analysis of variance (ANOVA) or χ^2^ test was used to compare basic characteristics among four quartile groups of serum albumin levels.

We used multivariable logistic regression models to examine the associations of serum albumin levels with ADL disability, mobility disability, and disability in objective physical functioning. The odds ratios (OR) and corresponding 95% confidence intervals (CI) were documented. The lowest quartile group of serum albumin levels was considered as the reference group and two models were considered. In model 1, we adjusted for age and sex. In model 2, we additionally adjusted for ethnicity, current marital status, residence, education, BMI, lifetime primary occupation, economic independence, being in receipt of adequate medication, co-residence with adult children, currently consuming alcohol, currently smoking, and regular exercise. In model 3, to determine the effect of serum vitamin D levels on associations of serum albumin with physical disability, we further adjusted for serum vitamin D levels based on model 2.

Next, we examined the association of serum albumin and disability in physical functioning by serum vitamin D status. The significance of the multiplication interaction between serum albumin and serum vitamin D levels was estimated by adding cross-product terms in models. We evaluated three indices and their 95% CI to determine if there was an additive interaction between serum albumin and vitamin D on physical functioning. The three indices included the interaction contrast ratio (ICR), attributable proportion due to interaction (AP), and the synergy index (S). If there was no statistically significant additive interaction, the 95% CI of ICR and AP included 0 and the 95% CI of S included 1 (47). In addition, restricted cubic splines (RCS) were performed to determine the Non-linear associations of serum albumin levels with ADL disability, mobility disability, and disability in objective physical functioning with four knots at the 5th, 25th, 75th, 95th of serum albumin levels distribution based on model 2.

To examine whether the associations of serum albumin with outcomes differed by age, sex, and the status of comorbidity, we repeated the main analyses by age (aged 65–79 years or ≥80 years), sex, and comorbidity with the same covariates adjusted in model 2. In addition, we conducted several additional analyses to assess the robustness of our results. First, in model 4, we repeated all analyses additionally adjusted for five chronic diseases (diabetes mellitus, hypertension, heart disease, stroke/cerebrovascular disease, and respiratory diseases) based on model 2. Second, in model 5, we repeated the main analysis further adjusting for MCI based on model 2. All analyses were performed using SAS version 9.4 (SAS Institute, Cary, NC). A P <0.05 was considered to be statistically significant.

## Results

### Basic Characteristics of Study Participants

The mean age (± SD) of study participants was 86.3 ± 12.2 years, and 55.1% (*N* = 1231) were females. Compared with participants with lower serum albumin levels (quartile 1), participants with higher serum albumin levels tended to be older, and more likely to be female, Han, residents of rural areas, to have a formal education, and to have more social contact and support (i.e., married and co-residence with children). Meanwhile, they were more likely to have a lower proportion of regular exercise, to consume alcohol, to have adequate medication, economic independence, and chronic diseases (hypertension, diabetes mellitus, and heart disease). Participants with higher serum albumin levels tended to have a higher BMI and higher serum vitamin D levels, compared with participants with lower serum albumin levels. Details about the characteristics of study participants are shown in Table 1.

**Table 1:** Basic characteristics of study participants by serum albumin quartiles.

**Characteristics**	**Overall (*N* = 2,233)**	**Serum albumin level (g/L)**				***P-*value**
		**<36.8 g/L^a^**	**36.8–40.1 g/L**	**40.1–43.3 g/L**	**>43.3 g/L**	
		**(*N* = 554)**	**(*N* = 571)**	**(*N* = 544)**	**(*N* = 564)**	
Age, mean ± SD, years,	86.3 ± 12.2	91.9 ± 10.8	88.0 ± 12.2	84.0 ± 12.1	81.3 ± 11.2	<0.001
Sex, female (%)	1,231 (55.1)	345 (62.3)	319 (55.9)	282 (51.8)	285 (50.5)	<0.001
Ethnicity, Han (%)	2,132 (95.5)	514 (92.8)	548 (96.0)	521 (95.8)	549 (97.3)	0.003
Residence, rural (%)	1,844 (82.6)	481 (86.8)	492 (86.2)	446 (82.0)	425 (75.4)	<0.001
≥1 years of education (%)	812 (36.4)	157 (28.3)	179 (31.3)	201 (36.9)	275 (48.8)	<0.001
White collar occupation (%)	46 (2.1)	9 (1.6)	7 (1.2)	12 (2.2)	18 (3.2)	0.106
Married, yes (%)	812 (36.4)	128 (23.1)	184 (32.2)	212 (39.0)	288 (51.1)	<0.001
Regular exercise, yes (%)	328 (14.7)	58 (10.5)	79 (13.8)	79 (14.5)	112 (19.9)	<0.001
Currently smoking, yes (%)	366 (16.4)	92 (16.6)	90 (15.8)	92 (16.9)	92 (16.3)	0.961
Currently consuming alcohol, yes (%)	329 (14.7)	58 (10.5)	80 (14.0)	88 (16.2)	103 (18.3)	0.002
BMI, mean ± SD, kg/m^2^	21.7 ± 12.7	20.7 ± 8.2	21.7 ± 19.6	21.5 ± 8.3	22.9 ± 11.0	0.037
Adequate medication, yes (%)	2,116 (94.8)	522 (94.2)	534 (93.5)	510 (93.8)	550 (97.5)	0.008
Co-residence with children, yes (%)	1,746 (78.2)	428 (77.3)	438 (76.7)	424 (77.9)	456 (80.9)	0.337
Economic independence, yes (%)	496 (22.2)	73 (13.2)	110 (19.3)	154 (28.3)	159 (28.2)	<0.001
serum vitamin D, mean ± SD,	41.7 ± 19.4	36.9 ± 17.9	40.8 ± 18.2	43.4 ± 19.8	45.5 ± 20.7	<0.001
Hypertension, yes (%)	588 (26.3)	115 (20.8)	164 (28.7)	147 (27.0)	162 (28.7)	0.006
Diabetes mellitus, yes (%)	49 (2.2)	9 (1.6)	10 (1.8)	12 (2.2)	18 (3.2)	0.264
Heart disease, yes (%)	167 (7.5)	27 (4.9)	35 (6.1)	44 (8.1)	61 (10.8)	0.001
Stroke/CVD, yes (%)	178 (8.0)	39 (7.0)	54 (9.5)	50 (9.2)	35 (6.2)	0.118
Respiratory diseases, yes (%)	190 (8.5)	45 (8.1)	52 (9.1)	47 (8.6)	46 (8.2)	0.925
MCI, yes	825 (36.9)	316 (57.0)	236 (41.3)	148 (27.2)	125 (22.2)	<0.001

*SD, standard deviation; BMI, body mass index; CVD, cardiovascular disease; MCI, mild cognitive impairment*.

*Values are given as No. (%) unless otherwise stated*.

a *The cutoff values of serum albumin levels were 36.8, 40.1, and 43.4 g/L*.

### Associations of Serum Albumin Level With Disability

The associations of serum albumin level with ADL disability, mobility disability, and disability in objective physical functioning are shown in Table 2. After adjusting for age and sex, we observed dose-response associations of serum albumin level with ADL disability, mobility disability, and disability in two objective tasks (i.e., standing up from a chair and picking up a book from the floor) (all P for trend <0.05). Compared with participants in the lowest quartile group of serum albumin level, those in the highest quartile group had 45% lower odds of ADL disability (OR: 0.55; 95% CI: 0.38, 0.80); 48% lower odds of mobility disability (OR: 0.52; 95% CI: 0.38, 0.71); 46% lower odds of disability in standing up from a chair (OR: 0.54; 95% CI: 0.34, 0.85); and 37% lower odds of disability in picking up a book from the floor (OR: 0.63; 95% CI: 0.40, 0.97). The findings were maintained after controlling for more covariates. For instance, the odds of ADL disability in the fully adjusted model was 0.50 (95% CI: 0.34, 0.74) for the highest group of serum albumin levels. Additionally, we observed a marginally significant association of serum albumin level with disability in turning around 360°. Compared with participants in the lowest quartile group of serum albumin levels, those in the highest quartile group had 27% lower odds of disability in turning around 360° (OR: 0.73; 95% CI: 0.51, 1.03). In model 3, after further adjusting for serum vitamin D, the statistically significant association of serum albumin with ADL disability, mobility disability, and disability in standing up from a chair were maintained. Although the associations of serum albumin with disability in picking up a book from the floor and turning around 360° did not achieve statistical significance, the potential protective effects of serum albumin on physical functioning were maintained. For instance, compared with participants in the lowest quartile group of serum albumin level, those in the highest quartile group had 34% lower odds of disability in picking up a book from the floor (OR: 0.66; 95% CI: 0.41, 1.06).

**Table 2:** Associations of serum albumin level with ADL disability, mobility disability, and disability in objective physical functioning.

	**No. of events/No. of participants**	**Model 1^a^**		**Model 2^b^**		**Model 3^c^**	
		**OR (95% CI)**	***P* for trend**	**OR (95% CI)**	***P* for trend**	**OR (95% CI)**	***P* for trend**
**ADL disability**	434/2,119						
<36.8 g/L^d^	176/554	Ref.		Ref.		Ref.	
36.8–40.1 g/L	122/571	0.72 (0.53, 0.97)	0.001	0.70 (0.51, 0.96)	<0.001	0.77 (0.56, 1.07)	0.001
40.1–43.3 g/L	79/544	0.63 (0.45, 0.88)		0.60 (0.42, 0.84)		0.67 (0.47, 0.96)	
>43.3 g/L	57/564	0.55 (0.38, 0.80)		0.50 (0.34, 0.74)		0.54 (0.36, 0.80)	
**Mobility disability**	1,057/2,188						
<36.8 g/L	360/554	Ref.		Ref.		Ref.	
36.8–40.1 g/L	298/571	0.79 (0.59, 1.06)	<0.001	0.72 (0.53, 0.98)	<0.001	0.72 (0.53, 0.98)	<0.001
40.1–43.3 g/L	233/544	0.84 (0.62, 1.14)		0.80 (0.58, 1.11)		0.81 (0.59, 1.12)	
>43.3 g/L	166/564	0.52 (0.38, 0.71)		0.51 (0.37, 0.71)		0.51 (0.37, 0.71)	
**Disability in objective physical functioning**							
**Standing up from a chair**	213/2,202						
<36.8 g/L	97/554	Ref.		Ref.		Ref.	
36.8–40.1 g/L	47/571	0.51 (0.34, 0.74)	0.004	0.49 (0.33, 0.73)	0.003	0.53 (0.35, 0.78)	0.009
40.1–43.3 g/L	40/544	0.61 (0.41, 0.92)		0.59 (0.38, 0.89)		0.63 (0.41, 0.96)	
>43.3 g/L	29/564	0.54 (0.34, 0.85)		0.52 (0.32, 0.84)		0.54 (0.33, 0.88)	
**Picking up a book from the floor**	258/2,194						
<36.8 g/L	111/554	Ref.		Ref.		Ref.	
36.8–40.1 g/L	66/571	0.64 (0.45, 0.91)	0.017	0.62 (0.43, 0.89)	0.012	0.69 (0.48, 1.00)	0.057
40.1–43.3 g/L	47/544	0.67 (0.45, 0.99)		0.63 (0.42, 0.94)		0.71 (0.47, 1.08)	
>43.3 g/L	34/564	0.63 (0.40, 0.97)		0.60 (0.38, 0.95)		0.66 (0.41, 1.06)	
**Turning around 360**°	521/2,211						
<36.8 g/L	199/554	Ref.		Ref.		Ref.	
36.8–40.1 g/L	138/571	0.69 (0.51, 0.92)	0.053	0.65 (0.48, 0.89)	0.048	0.69 (0.51, 0.94)	0.102
40.1–43.3 g/L	101/544	0.71 (0.52, 0.98)		0.67 (0.49, 0.94)		0.72 (0.52, 1.00)	
>43.3 g/L	83/564	0.74 (0.53, 1.03)		0.73 (0.51, 1.03)		0.76 (0.53, 1.08)	

*ADL, activities of daily living; OR, odds ratio; CI, confidence interval*.

a *Model 1 adjusted for age and sex*.

b *Based on model 1, model 2 further adjusted for ethnicity, residence, current marital status, education, lifetime primary occupation, economic independence, being in receipt of adequate medication, co-residence with adult children, currently smoking, currently consuming alcohol, BMI, regular exercise*.

c *Based on model 2, model 3 further adjusted for serum vitamin D levels*.

d *The cutoff values of serum albumin levels were 36.8, 40.1, and 43.4 g/L*.

### Additional Analyses

**Figure 2:**
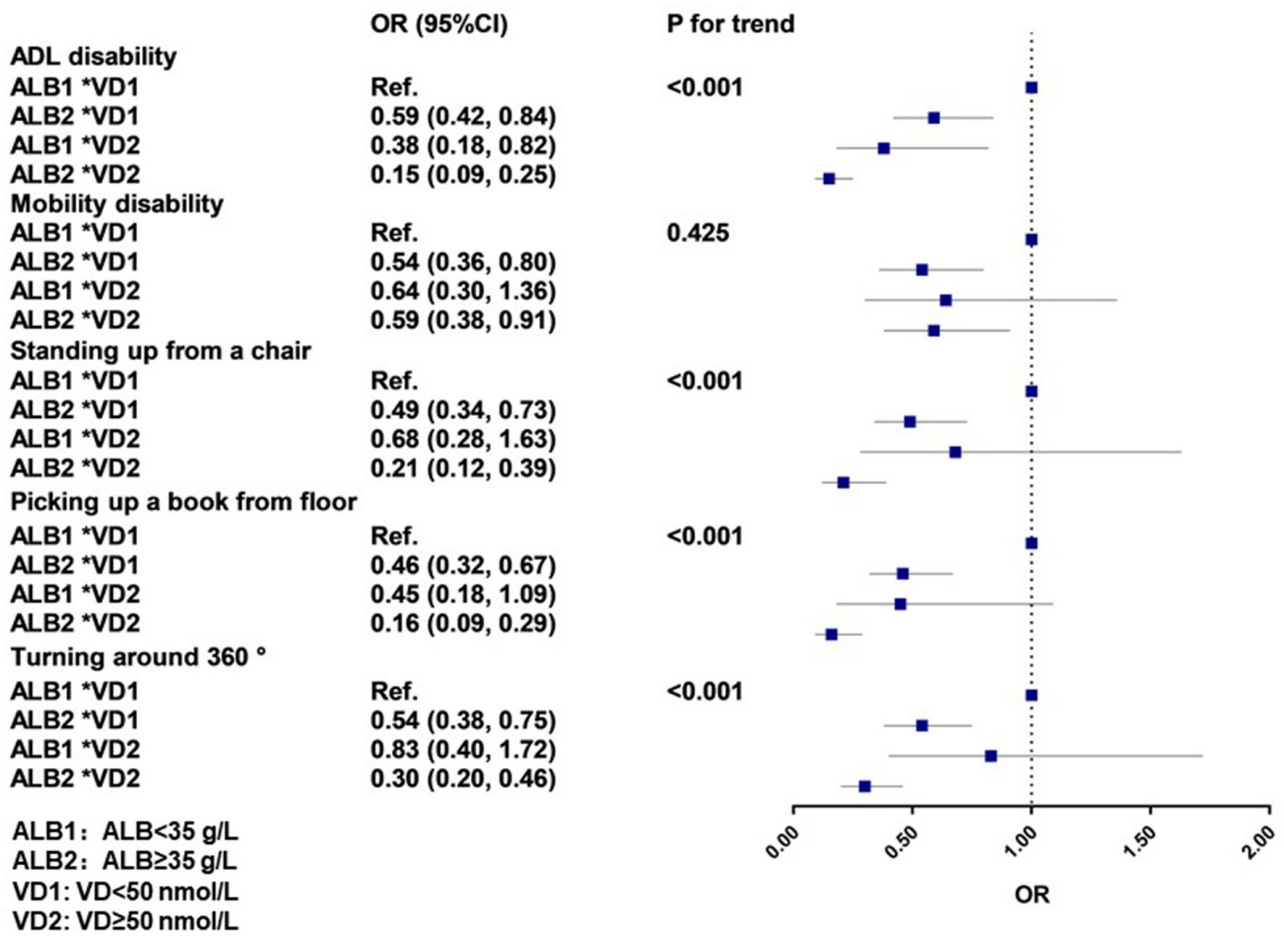
Joint associations of serum albumin level and vitamin D with disability in physical functioning. OR, odds ratio; CI, confidence interval; ALB, albumin; VD, vitamin D; ADL, activities of daily living. The cutoff value was 35 g/L and 50 nmol/L for serum albumin and serum vitamin D level, respectively. ALB1 represents serum albumin levels of the participants were below 35 g/L (<35 g/L), ALB2 represents serum albumin levels of the participants were above 35 (≥35 g/L). VD1 represents the serum vitamin D level of the participants were below 50 (<50 nmol/L), VD2 represents the serum vitamin D level of the participants were above 50 (≥50 nmol/L). In this model, based on model 1, we further adjusted for ethnicity, residence, current marital status, education, lifetime primary occupation, economic independence, being in receipt of adequate medication, co-residence with adult children, currently smoking, currently consuming alcohol, BMI, and regular exercise.

Figure 2 shows the associations of serum albumin levels and disability in physical functioning by serum vitamin D level. We did not observe the statistically significant multiplication (data not shown) or additive interaction effects (Supplementary Table 1) between serum albumin and vitamin D level on disability in physical functioning (all P for interaction > 0.05) in our analyses, although participants with a combination of higher serum albumin levels and serum vitamin D levels had the lowest odds of disability in physical functioning. For instance, relative to participants with a combination of lower serum albumin levels and serum vitamin D levels, those with a combination of higher serum albumin levels and serum vitamin D levels had 85% lower odds of ADL disability (OR: 0.15; 95% CI: 0.09, 0.25); 79% lower odds of disability in standing up from a chair (OR: 0.21; 95% CI: 0.12, 0.39); 84% lower odds of disability in picking up a book from the floor (OR: 0.16; 95% CI: 0.09, 0.29); and 70% lower risk of disability in turning around 360° (OR: 0.30; 95% CI: 0.19, 0.46). Similarly, the association of serum albumin level with disability in physical functioning by serum vitamin D level was maintained after further adjustment for five chronic diseases (Supplementary Table 2).

Supplementary Figure 1 presents the Non-linear associations of serum albumin level with ADL disability, mobility disability, and disability in objective physical functioning. We observed that with the increase of serum albumin levels, the OR of disability in physical functioning was decreasing within the serum albumin levels of roughly <52.5 g/L. For ADL disability and mobility disability, we found a linear association (*P* for nonlinear = 0.059 and 0.339, respectively) when treating serum albumin levels as continuous variables. The results of stratified analyses by age, sex, and comorbidity were shown in Supplementary Tables 3–5, respectively. No statistically significant interactions between age, sex, and comorbidity and serum albumin level on disability in physical functioning were found, with one exception (i.e., serum albumin and sex on disability in turning around 360°). In model 4, after further adjusting for five chronic diseases, the statistically significant associations of serum albumin levels with disability in physical functioning remained. For instance, compared with participants in the lowest quartile group of serum albumin levels, those in the highest quartile group had lower odds of disability in ADL disability, mobility disability, disability in standing up from a chair, and disability in picking up a book from the floor, with ORs of 0.51 (95% CI: 0.34, 0.76), 0.51 (95% CI: 0.37, 0.72), 0.54 (95% CI: 0.33, 0.88), 0.66 (95% CI: 0.41, 1.06), respectively. In model 5, after further adjustment for MCI based on model 2, the protective effect of higher serum albumin on physical functioning was maintained, though the associations between serum albumin levels and a few outcomes such as disability in picking up a book from the floor did not achieve the statistical significance (Supplementary Table 6).

## Discussion

In this study, we observed statistically significant associations between serum albumin level and various indicators of disability in physical functioning among Chinese older adults. These associations were regardless of vitamin D level since no interaction between serum albumin and vitamin D level on disability in physical functioning was observed in our study. These findings suggest that serum albumin level could be a potentially useful biomarker of disability in physical functioning among older adults.

Our findings of the associations between serum albumin level and disability in physical functioning are consistent with previous studies (8–13, 22, 48–53). Notably, one study observed that there was no association of serum albumin with the decline in functional performance in a 3 years follow-up study (54). In addition to differences in measurements of physical functioning included (chair stand, putting on and taking off a cardigan, 3-meter walk), the disparities may be attributed to the difference in characteristics of the study population. Regarding the association of a combined lower serum albumin and vitamin D level with disability in physical functioning, our results are consistent with a study performed in Japan (22). Considering the findings of previous studies that vitamin D was also associated with the decline in physical function (14, 55), we hypothesize that there would be a joint effect of a low level of serum albumin and vitamin D on the risk of disability in physical functioning. However, we did not observe a multiplication or additive interaction between serum albumin and vitamin D on physical disability, even though participants with a combination of lower serum albumin and vitamin D level had high odds of physical disability in our study. We speculate that serum albumin and vitamin D may play a different role in the process of functional decline.

The mechanisms explaining the observed association between serum albumin and disability in physical functioning are not clear. A previous study demonstrated that a lower serum albumin level was associated with poor nutritional status among older adults aged above 90 years old (56). Due to the poor nutritional status of older adults, the lack of serum proteins needed for muscle synthesis and strength maintenance might partially contribute to the decline in physical functioning. Simultaneously, a relatively low level of serum albumin *per se* reflects an unhealthy status. Previous studies demonstrated that serum albumin was associated with many diseases such as diabetes (57) and metabolic syndrome (58). Thus, we further adjusted for five main chronic diseases to control for the possible confounding effect of diseases. The results were maintained, emphasizing the potential of serum albumin as a biomarker of decline in physical functioning. In addition, researchers uncovered that serum albumin treatment reduced systemic inflammation in patients with decompensated cirrhosis (59). We speculate that a low serum albumin level reduces the ability of the body to resist inflammatory damage, a significant biological component of aging (60). Our findings indicate that appropriate management of poor nutritional status, in particular low serum albumin levels, may contribute to maintaining physical functioning in older adults. Recently, randomized controlled trials found that multimodal nutritional intervention significantly improved the physical functioning of older adults (14, 61). Although clinical trials directly targeting serum albumin have not been conducted, it is believed that such investigations would be of much value.

The main strengths of our study include the use of data from a large sample from a national survey of Chinese older adults and a relatively comprehensive assessment of physical functioning with a combination of objective and subjective measurements. In addition, the consistency of the associations between serum albumin level and these multiple indicators of physical functioning verified the robustness of our results. Nevertheless, this study has several limitations. First, an inevitable limitation is the cross-sectional design of our study, which does not allow for causal inference. Second, it is the lack of information on daily nutrition intake that may confound the findings. Third, since the level of serum albumin is dynamic, only one assessment of serum albumin level may result in some measurement errors as it may not reflect the intra-person variability and long-term average level. Forth, considering that the rural population accounts for 82.6%, our findings may not be generalizable to other cases, e.g., when having a high proportion of urban older adults. Fifth, considering that 87.1% of the participants with serum albumin levels above 35 g/L and most of the participants in our dataset are relatively healthy, our findings may only provide instruction for relatively healthy older adults to manage nutrition status for preventing physical disability. Finally, our result may not be generalizable older adults in other countries.

In conclusion, we found that serum albumin level was associated with disability in physical functioning regardless of vitamin D level among Chinese older adults. Serum albumin level has the potential to serve as a useful biomarker of decline in physical functioning among older adults. The findings highlight the potential of preventing functional decline by managing serum albumin levels in older adults.

## Data Availability Statement

The data that support the findings of this study are available from the corresponding author Zuyun Liu, upon reasonable request.

## Ethics Statement

The studies involving human participants were reviewed and approved by the Duke University and the Research Ethics Committees of Peking University granted approval for the Protection of Human Subjects for the CLHLS and all participants provided informed consent. The patients/participants provided their written informed consent to participate in this study. Written informed consent was obtained from the individual(s) for the publication of any potentially identifiable images or data included in this article.

## Author Contributions

XL: methodology, data curation, software, visualization, writing—original draft preparation, and writing—review and editing. XC: methodology, data curation, software, writing—original draft preparation, and writing—review and editing. ZY: investigation, methodology, and writing—review and editing. JZ, XS, and EH: methodology and writing—review and editing. ZL: conceptualization, methodology, data curation, software, visualization, and writing—review and editing. All authors contributed to the article and approved the submitted version.

## Funding

This study was supported by a grant from the National Natural Science Foundation of China (Grant No. 82171584), and the 2020 Milstein Medical Asian American Partnership Foundation Irma and Paul Milstein Program for Senior Health project award (ZL), the Fundamental Research Funds for the Central Universities (ZL), a project from the Natural Science Foundation of Zhejiang Province (Grant No. LQ21H260003), Alibaba Cloud, Key Laboratory of Intelligent Preventive Medicine of Zhejiang Province (Grant No. 2020E10004), and Zhejiang University Global Partnership Fund (Grant No. 188170–11103). The data used in this study were from the Chinese Longitudinal Healthy Longevity Survey (CLHLS), which is managed by the Center for Healthy Aging and Development Studies, Peking University. The CLHLS was supported by funds from the U.S. National Institutes on Aging (Grant No. R01AG023627), China National Natural Science Foundation (Grant Nos. 71233001 and 71110107025), China Social Science Foundation, and UNFPA. The funders had no role in the study design; data collection, analysis, or interpretation; in the writing of the report; or in the decision to submit the article for publication.

## Conflict of Interest

The authors declare that the research was conducted in the absence of any commercial or financial relationships that could be construed as a potential conflict of interest.

## Publisher's Note

All claims expressed in this article are solely those of the authors and do not necessarily represent those of their affiliated organizations, or those of the publisher, the editors and the reviewers. Any product that may be evaluated in this article, or claim that may be made by its manufacturer, is not guaranteed or endorsed by the publisher.
