# Supplementary material for: Associations of Serum Albumin With Disability in Activities of Daily Living, Mobility and Objective Physical Functioning Regardless of Vitamin D: Cross-Sectional Findings From the Chinese Longitudinal Healthy Longevity Survey
Source: Front Nutr. 2022 Feb 24;9:809499. doi: 10.3389/fnut.2022.809499 (PMC8908380; doi:10.3389/fnut.2022.809499)
Supplement: Supplementary file 1 [file Data_Sheet_1.docx]

**Table S1.** **Additive interaction between serum albumin and vitamin D in associations with disability in physical functioning.**

Abbreviations: ADL, activities of daily living; ICR: interaction contrast ratio; AP: attributable proportion due to interaction; S: synergy index.

|  | **Estimate (95%CI)** | **P value** |
| --- | --- | --- |
| **ADL disability** |  |  |
| ICR | -0.02 (-1.11, 1.07) | 0.971 |
| AP | -0.09 (-4.98, 4.79) | 0.970 |
| S | -0.63 (-1.64, 0.37) | 0.217 |
| **Mobility disability** |  |  |
| ICR | 0.62 (0.25, 0.99) | 0.001 |
| AP | 7.66 (-7.78, 23.1) | 0.331 |
| S | -1.98 (-4.30, 0.33) | 0.093 |
| **Disability in objective physical functioning** | | |
| **Standing up from a chair** |  |  |
| ICR | -0.32 (-2.09, 1.44) | 0.719 |
| AP | -0.92 (-4.25, 2.41) | 0.589 |
| S | -0.39 (-1.33, 0.56) | 0.424 |
| **Picking up a book from the floor** |  |  |
| ICR | -0.01 (-1.32, 1.30) | 0.987 |
| AP | -0.05 (-5.89, 5.79) | 0.986 |
| S | -0.63 (-1.83, 0.57) | 0.302 |
| **Turning around 360**° |  |  |
| ICR | -0.11 (-1.22, 0.99) | 0.840 |
| AP | -0.36 (-3.38, 2.65) | 0.814 |
| S | -0.48 (-1.31, 0.35) | 0.257 |

**Table S2**. **Joint associations of serum albumin and vitamin D with disability in physical functioning.**

|  | **No. of events/No. of participants** | **OR (95%CI)** | **P for trend** |
| --- | --- | --- | --- |
|  |  |  |  |
| **ADL disability** | 434/2108 |  |  |
| ALB1*VD1 | 93/208 | Ref. | <0.001 |
| ALB2*VD1 | 298/1264 | 0.57 (0.40, 0.81) |  |
| ALB1*VD2 | 12/58 | 0.38 (0.17, 0.83) |  |
| ALB2*VD2 | 31/578 | 0.15 (0.09, 0.25) |  |
| **Mobility disability** | 1057/2176 |  |  |
| ALB1*VD1 | 164/217 | Ref. | 0.479 |
| ALB2*VD1 | 641/1312 | 0.54 (0.36, 0.80) |  |
| ALB1*VD2 | 32/58 | 0.64 (0.30, 1.35) |  |
| ALB2*VD2 | 216/589 | 0.60 (0.38, 0.93) |  |
| **Standing up from a chair** | 213/2191 |  |  |
| ALB1*VD1 | 53/221 | Ref. | <0.001 |
| ALB2*VD1 | 135/1324 | 0.47 (0.32, 0.70) |  |
| ALB1*VD2 | 8/58 | 0.70 (0.29, 1.72) |  |
| ALB2*VD2 | 17/588 | 0.21 (0.11, 0.39) |  |
| **Picking up a book from the floor** | 258/2182 |  |  |
| ALB1*VD1 | 66/221 | Ref. | <0.001 |
| ALB2*VD1 | 166/1325 | 0.44 (0.31, 0.65) |  |
| ALB1*VD2 | 8/57 | 0.44 (0.18, 1.09) |  |
| ALB2*VD2 | 18/579 | 0.16 (0.09, 0.29) |  |
| **Turning around 360**° | 521/2199 |  |  |
| ALB1*VD1 | 104/218 | Ref. | <0.001 |
| ALB2*VD1 | 332/1338 | 0.52 (0.37, 0.73) |  |
| ALB1*VD2 | 19/56 | 0.85 (0.41, 1.79) |  |
| ALB2*VD2 | 63/587 | 0.30 (0.19, 0.46) |  |

Abbreviations: OR, odds ratio; CI, confidence interval; ALB, albumin; VD, vitamin D; ADL, activities of daily living. The cutoff value was 35 g/L and 50 nmol/L for serum albumin and serum vitamin D level, respectively. ALB1 represents serum albumin levels of the participants were below 35 g/L (<35 g/L), ALB2 represents serum albumin levels of the participants were above 35 (≥35 g/L). VD1 represents serum vitamin D level of the participants were below 50 (<50 nmol/L), VD1 represents serum vitamin D level of the participants were above 50 (≥50 nmol/L).

In this model, based on model 1, we further adjusted for ethnicity, residence, current marital status, education, lifetime primary occupation, economic independence, being in receipt of adequate medication, co-residence with adult children, currently smoking, currently consuming alcohol, BMI, regular exercise, and five chronic diseases (diabetes mellitus, hypertension, heart disease, stroke/cerebrovascular disease, and respiratory diseases).

**Table S3**. **Associations of serum albumin level with ADL disability, mobility disability, and disability in objective physical functioning stratified by age.**

|  | **No. of events/No. of participants** | **Model 2 ^a^** | **P for trend** |
| --- | --- | --- | --- |
|  |  | **OR (95%CI)** |  |
| **ADL disability** | 434/2119 |  |  |
| **65-79 years** | 18/700 |  |  |
| <36.8 g/L ^b^ | 4/81 | Ref. |  |
| 36.8-40.1 g/L | 4/150 | 0.50 (0.12, 2.13) | 0.119 |
| 40.1-43.4 g/L | 6/203 | 0.60 (0.15, 2.34) |  |
| >43.4 g/L | 4/266 | 0.27 (0.06, 1.19) |  |
| **≥80 years** | 416/1419 |  |  |
| <36.8 g/L | 172/444 | Ref. |  |
| 36.8-40.1 g/L | 118/393 | 0.72 (0.52, 0.99) | 0.001 |
| 40.1-43.4 g/L | 73/311 | 0.59 (0.41, 0.85) |  |
| >43.4 g/L | 53/271 | 0.54 (0.36, 0.81) |  |
| Interaction P value: ALB × Age group |  | 0.390 |  |
| **Mobility disability** | 1057/2188 |  |  |
| **65-79 years** | 95/720 |  |  |
| <36.8 g/L | 11/83 | Ref. |  |
| 36.8-40.1 g/L | 24/158 | 1.17 (0.51, 2.66) | 0.902 |
| 40.1-43.4 g/L | 31/209 | 1.38 (0.62, 3.10) |  |
| >43.4 g/L | 29/270 | 0.90 (0.40, 2.00) |  |
| **≥80 years** | 962/1468 |  |  |
| <36.8 g/L | 349/462 | Ref. |  |
| 36.8-40.1 g/L | 274/409 | 0.66 (0.47, 0.93) | <0.001 |
| 40.1-43.4 g/L | 202/318 | 0.74 (0.51, 1.06) |  |
| >43.4 g/L | 137/279 | 0.44 (0.31, 0.64) |  |
| Interaction P value: ALB × Age group |  | 0.321 |  |
| **Disability in objective physical functioning** | | | |
| **Standing up from a chair** | 213/2202 |  |  |
| **65-79 years** | 17/714 |  |  |
| <36.8 g/L | 3/83 | Ref. |  |
| 36.8-40.1 g/L | 1/156 | 0.18 (0.02, 1.81) | 0.239 |
| 40.1-43.4 g/L | 4/207 | 0.58 (0.12, 2.74) |  |
| >43.4 g/L | 9/268 | 1.16 (0.29, 4.67) |  |
| **≥80 years** | 196/1488 |  |  |
| <36.8 g/L | 94/462 | Ref. |  |
| 36.8-40.1 g/L | 46/411 | 0.51 (0.34, 0.77) | 0.001 |
| 40.1-43.4 g/L | 36/327 | 0.60 (0.39, 0.94) |  |
| >43.4 g/L | 20/288 | 0.42 (0.24, 0.72) |  |
| Interaction P value: ALB × Age group |  | 0.192 |  |
| **Picking up a book from the floor** | 258/2194 |  |  |
| **65-79 years** | 7/708 |  |  |
| <36.8 g/L | 2/83 | Ref. |  |
| 36.8-40.1 g/L | 2/151 | 0.64 (0.08, 4.97) | 0.286 |
| 40.1-43.4 g/L | 1/208 | 0.22 (0.02, 2.63) |  |
| >43.4 g/L | 2/266 | 0.32 (0.04, 2.54) |  |
| **≥80 years** | 251/1486 |  |  |
| <36.8 g/L | 109/463 | Ref. |  |
| 36.8-40.1 g/L | 64/412 | 0.62 (0.43, 0.89) | 0.022 |
| 40.1-43.4 g/L | 46/327 | 0.66 (0.44, 0.99) |  |
| >43.4 g/L | 32/284 | 0.62 (0.39, 0.99) |  |
| Interaction P value: ALB × Age group |  | 0.152 |  |
| **Turning around 360**° | 521/2211 |  |  |
| **65-79 years** |  |  |  |
| <36.8 g/L | 202/556 | Ref. |  |
| 36.8-40.1 g/L | 135/552 | 0.28 (0.06, 1.28) | 0.739 |
| 40.1-43.4 g/L | 101/547 | 0.31 (0.08, 1.26) |  |
| >43.4 g/L | 83/556 | 0.54 (0.16, 1.83) |  |
| **≥80 years** |  |  |  |
| <36.8 g/L | 180/540 | Ref. |  |
| 36.8-40.1 g/L | 118/528 | 0.67 (0.49, 0.91) | 0.065 |
| 40.1-43.4 g/L | 79/519 | 0.71 (0.51, 1.00) |  |
| >43.4 g/L | 57/532 | 0.72 (0.50, 1.04) |  |
| Interaction P value: ALB × Age group |  | 0.900 |  |

Abbreviations: ADL, activities of daily living; ALB, albumin; OR, odds ratio; CI, confidence interval.

^a^ Model 2 adjusted for age, sex, ethnicity, residence, current marital status, education, lifetime primary occupation, economic independence, being in receipt of adequate medication, co-residence with adult children, currently smoking, currently consuming alcohol, BMI, regular exercise.

^b^ The cutoff values were 36.8, 40.1, and 43.4 g/L.

**Table S4**. **Associations of serum albumin level with ADL disability, mobility disability, and disability in objective physical functioning stratified by sex.**

|  | **No. of events/No. of participants** | **Model 2 ^a^** |  |
| --- | --- | --- | --- |
|  |  | **OR (95%CI)** | **P for trend** |
| **ADL disability** | 434/2119 |  |  |
| **Women** | 320/1164 |  |  |
| <36.8 g/L ^b^ | 134/326 | Ref. |  |
| 36.8-40.1 g/L | 95/301 | 0.77 (0.53, 1.12) | 0.002 |
| 40.1-43.4 g/L | 52/267 | 0.56 (0.37, 0.86) |  |
| >43.4 g/L | 39/270 | 0.53 (0.33, 0.85) |  |
| **Men** | 114/955 |  |  |
| <36.8 g/L | 42/199 | Ref. |  |
| 36.8-40.1 g/L | 27/242 | 0.51 (0.28, 0.92) | 0.015 |
| 40.1-43.4 g/L | 27/247 | 0.61 (0.34, 1.12) |  |
| >43.4 g/L | 18/267 | 0.40 (0.20, 0.79) |  |
| Interaction P value: ALB ×Sex group |  | 0.978 |  |
| **Mobility disability** | 1057/2188 |  |  |
| **Women** | 744/1204 |  |  |
| <36.8 g/L | 257/343 | Ref. |  |
| 36.8-40.1 g/L | 212/317 | 0.89 (0.59, 1.35) | 0.186 |
| 40.1-43.4 g/L | 163/270 | 1.18 (0.76, 1.84) |  |
| >43.4 g/L | 112/274 | 0.65 (0.42, 1.01) |  |
| **Men** | 313/984 |  |  |
| <36.8 g/L | 103/202 | Ref. |  |
| 36.8-40.1 g/L | 86/250 | 0.55 (0.35, 0.88) | <0.001 |
| 40.1-43.4 g/L | 70/257 | 0.51 (0.32, 0.82) |  |
| >43.4 g/L | 54/275 | 0.37 (0.23, 0.60) |  |
| Interaction P value: ALB × Sex group |  | 0.069 |  |
| **Disability in objective physical functioning** | |  |  |
| **Standing up from a chair** | 213/2202 |  |  |
| **Women** | 160/1218 |  |  |
| <36.8 g/L | 79/343 | Ref. |  |
| 36.8-40.1 g/L | 36/317 | 0.47 (0.30, 0.75) | 0.003 |
| 40.1-43.4 g/L | 24/279 | 0.46 (0.27, 0.78) |  |
| >43.4 g/L | 21/279 | 0.50 (0.28, 0.89) |  |
| **Men** | 53/984 |  |  |
| <36.8 g/L | 18/202 | Ref. |  |
| 36.8-40.1 g/L | 11/250 | 0.55 (0.25, 1.23) | 0.464 |
| 40.1-43.4 g/L | 16/255 | 0.98 (0.46, 2.09) |  |
| >43.4 g/L | 8/277 | 0.55 (0.22, 1.39) |  |
| Interaction P value: ALB × Sex group |  | 0.236 |  |
| **Picking up a book from the floor** | 258/2194 |  |  |
| **Women** | 197/1213 |  |  |
| <36.8 g/L | 85/341 | Ref. |  |
| 36.8-40.1 g/L | 51/315 | 0.66 (0.43, 1.00) | 0.149 |
| 40.1-43.4 g/L | 33/279 | 0.66 (0.41, 1.06) |  |
| >43.4 g/L | 28/278 | 0.76 (0.45, 1.29) |  |
| **Men** | 61/981 |  |  |
| <36.8 g/L | 26/205 | Ref. |  |
| 36.8-40.1 g/L | 15/248 | 0.49 (0.24, 1.01) | 0.016 |
| 40.1-43.4 g/L | 14/256 | 0.57 (0.27, 1.21) |  |
| >43.4 g/L | 6/272 | 0.28 (0.10, 0.76) |  |
| Interaction P value: ALB × Sex group |  | 0.187 |  |
| **Turning around 360**° | 521/2211 |  |  |
| **Women** | 407/1219 |  |  |
| <36.8 g/L | 150/335 | Ref. |  |
| 36.8-40.1 g/L | 108/318 | 0.73 (0.51, 1.05) | 0.672 |
| 40.1-43.4 g/L | 76/281 | 0.78 (0.52, 1.16) |  |
| >43.4 g/L | 73/285 | 1.18 (0.77, 1.81) |  |
| **Men** | 114/992 |  |  |
| <36.8 g/L | 49/205 | Ref. |  |
| 36.8-40.1 g/L | 30/250 | 0.46 (0.26, 0.82) | <0.001 |
| 40.1-43.4 g/L | 25/260 | 0.46 (0.25, 0.83) |  |
| >43.4 g/L | 10/277 | 0.17 (0.08, 0.37) |  |
| Interaction P value: ALB × Sex group |  | <0.001 |  |

Abbreviations: ADL, activities of daily living; ALB, albumin; OR, odds ratio; CI, confidence interval.

^a^ Model 2 adjusted for age, sex, ethnicity, residence, current marital status, education, lifetime primary occupation, economic independence, being in receipt of adequate medication, co-residence with adult children, currently smoking, currently consuming alcohol, BMI, and regular exercise.

^b^ The cutoff values of serum albumin level were 36.8, 40.1, and 43.4 g/L.

**Table S5**. **Associations of serum albumin level with ADL disability, mobility disability, and disability in objective physical functioning stratified by with or without comorbidity.**

|  | **No. of events/No. of participants** | **Model 2 ^a^** | **P for trend** |
| --- | --- | --- | --- |
|  |  | **OR (95%CI)** |  |
| **ADL disability** | 434/2119 |  |  |
| **With comorbidity** | 76/310 |  |  |
| <36.8 g/L ^b^ | 24/71 | Ref. |  |
| 36.8-40.1 g/L | 24/87 | 0.78 (0.35, 1.72) | 0.154 |
| 40.1-43.4 g/L | 18/82 | 0.74 (0.31, 1.77) |  |
| >43.4 g/L | 10/70 | 0.47 (0.18, 1.26) |  |
| **Without comorbidity** | 358/1809 |  |  |
| <36.8 g/L | 152/454 | Ref. |  |
| 36.8-40.1 g/L | 98/456 | 0.66 (0.47, 0.93) | <0.001 |
| 40.1-43.4 g/L | 61/432 | 0.53 (0.36, 0.78) |  |
| >43.4 g/L | 47/467 | 0.49 (0.32, 0.75) |  |
| Interaction P value: ALB × Comorbidity group |  | 0.279 |  |
| **Mobility disability** | 1057/2188 |  |  |
| **With comorbidity** | 154/325 |  |  |
| <36.8 g/L | 47/79 | Ref. |  |
| 36.8-40.1 g/L | 46/89 | 0.81 (0.37, 1.75) | 0.399 |
| 40.1-43.4 g/L | 34/85 | 0.70 (0.30, 1.60) |  |
| >43.4 g/L | 27/72 | 0.71 (0.31, 1.63) |  |
| **Without comorbidity** | 903/1863 |  |  |
| <36.8 g/L | 313/466 | Ref. |  |
| 36.8-40.1 g/L | 252/478 | 0.70 (0.50, 0.99) | <0.001 |
| 40.1-43.4 g/L | 199/442 | 0.80 (0.56, 1.14) |  |
| >43.4 g/L | 139/477 | 0.48 (0.33, 0.68) |  |
| Interaction P value: ALB ×Comorbidity group |  | 0.168 |  |
| **Disability in objective physical functioning** | |  |  |
| **Standing up from a chair** | 213/2202 |  |  |
| **With comorbidity** | 42/328 |  |  |
| <36.8 g/L | 18/77 | Ref. |  |
| 36.8-40.1 g/L | 8/89 | 0.30 (0.11, 0.80) | 0.125 |
| 40.1-43.4 g/L | 12/87 | 0.66 (0.25, 1.75) |  |
| >43.4 g/L | 4/75 | 0.32 (0.09, 1.14) |  |
| **Without comorbidity** | 171/1874 |  |  |
| <36.8 g/L | 79/468 | Ref. |  |
| 36.8-40.1 g/L | 39/478 | 0.52 (0.34, 0.81) | 0.012 |
| 40.1-43.4 g/L | 28/447 | 0.54 (0.33, 0.87) |  |
| >43.4 g/L | 25/481 | 0.58 (0.35, 0.98) |  |
| Interaction P value: ALB ×Comorbidity group |  | 0.677 |  |
| **Picking up a book from the floor** | 258/2194 |  |  |
| **With comorbidity** | 48/332 |  |  |
| <36.8 g/L | 20/78 | Ref. |  |
| 36.8-40.1 g/L | 13/90 | 0.47 (0.20, 1.12) | 0.028 |
| 40.1-43.4 g/L | 11/89 | 0.52 (0.20, 1.38) |  |
| >43.4 g/L | 4/75 | 0.23 (0.06, 0.85) |  |
| **Without comorbidity** | 210/1862 |  |  |
| <36.8 g/L | 91/468 | Ref. |  |
| 36.8-40.1 g/L | 53/473 | 0.62 (0.41, 0.92) | 0.073 |
| 40.1-43.4 g/L | 36/446 | 0.63 (0.40, 1.00) |  |
| >43.4 g/L | 30/475 | 0.70 (0.43, 1.16) |  |
| Interaction P value: ALB ×Comorbidity group |  | 0.354 |  |
| **Turning around 360**° | 521/2211 |  |  |
| **With comorbidity** | 87/331 |  |  |
| <36.8 g/L | 31/75 | Ref. |  |
| 36.8-40.1 g/L | 26/90 | 0.51 (0.23, 1.15) | 0.058 |
| 40.1-43.4 g/L | 18/90 | 0.41 (0.17, 1.01) |  |
| >43.4 g/L | 12/76 | 0.42 (0.17, 1.09) |  |
| **Without comorbidity** | 434/1880 |  |  |
| <36.8 g/L | 168/465 | Ref. |  |
| 36.8-40.1 g/L | 112/478 | 0.66 (0.47, 0.92) | 0.159 |
| 40.1-43.4 g/L | 83/451 | 0.71 (0.49, 1.01) |  |
| >43.4 g/L | 71/486 | 0.78 (0.53, 1.15) |  |
| Interaction P value: ALB ×Comorbidity group |  | 0.307 |  |

Abbreviations: ADL, activities of daily living; ALB, albumin; OR, odds ratio; CI, confidence interval.

^a^ Model 2 adjusted for age, sex, ethnicity, residence, current marital status, education, lifetime primary occupation, economic independence, being in receipt of adequate medication, co-residence with adult children, currently smoking, currently consuming alcohol, BMI, and regular exercise.

^b^ The cutoff values of serum albumin level were 36.8, 40.1, and 43.4 g/L.

**Table S6. Associations of serum albumin level** **with ADL disability, mobility disability, and disability in objective physical functioning.**

|  | **No. of events/No. of participants** | **Model 4 ^a^** | **P for trend** | **Model 5 ^b^** | **P for trend** |
| --- | --- | --- | --- | --- | --- |
|  |  | **OR (95%CI)** |  | **OR (95%CI)** |  |
| **ADL disability** | 434/2119 |  |  |  |  |
| <36.8 g/L ^c^ | 180/540 | Ref. |  | Ref. |  |
| 36.8-40.1 g/L | 118/528 | 0.73 (0.53, 1.02) | 0.001 | 0.81 (0.58, 1.12) | 0.020 |
| 40.1-43.4 g/L | 79/519 | 0.64 (0.44, 0.92) |  | 0.75 (0.52, 1.09) |  |
| >43.4 g/L | 57/532 | 0.51 (0.34, 0.76) |  | 0.63 (0.42, 0.94) |  |
| **Mobility disability** | 1057/2188 |  |  |  |  |
| <36.8 g/L | 369/561 | Ref. |  | Ref. |  |
| 36.8-40.1 g/L | 289/551 | 0.71 (0.52, 0.98) | 0.001 | 0.77 (0.56, 1.05) | 0.005 |
| 40.1-43.4 g/L | 235/533 | 0.81 (0.59, 1.12) |  | 0.91 (0.65, 1.26) |  |
| >43.4 g/L | 164/543 | 0.51 (0.37, 0.72) |  | 0.57 (0.41, 0.80) |  |
| **Disability in objective physical functioning** | |  |  |  |  |
| **Standing up from a chair** | 213/2202 |  |  |  |  |
| <36.8 g/L | 100/561 | Ref. |  | Ref. |  |
| 36.8-40.1 g/L | 44/551 | 0.49 (0.33, 0.74) | 0.008 | 0.53 (0.35, 0.79) | 0.063 |
| 40.1-43.4 g/L | 40/540 | 0.60 (0.39, 0.93) |  | 0.71 (0.46, 1.10) |  |
| >43.4 g/L | 29/550 | 0.54 (0.33, 0.88) |  | 0.65 (0.39, 1.07) |  |
| **Picking up a book from the floor** | 258/2194 |  |  |  |  |
| <36.8 g/L | 114/562 | Ref. |  | Ref. |  |
| 36.8-40.1 g/L | 63/547 | 0.66 (0.45, 0.96) | 0.054 | 0.67 (0.46, 0.98) | 0.212 |
| 40.1-43.4 g/L | 47/541 | 0.70 (0.46, 1.06) |  | 0.75 (0.49, 1.16) |  |
| >43.4 g/L | 34/544 | 0.66 (0.41, 1.06) |  | 0.79 (0.48, 1.28) |  |
| **Turning around 360**° | 521/2211 |  |  |  |  |
| <36.8 g/L | 202/556 | Ref. |  | Ref. |  |
| 36.8-40.1 g/L | 135/552 | 0.66 (0.48, 0.90) | 0.101 | 0.72 (0.52, 0.98) | 0.521 |
| 40.1-43.4 g/L | 101/547 | 0.71 (0.50, 0.99) |  | 0.82 (0.58, 1.16) |  |
| >43.4 g/L | 83/556 | 0.75 (0.52, 1.08) |  | 0.89 (0.61, 1.29) |  |

Abbreviations: ADL, activities of daily living; OR, odds ratio; CI, confidence interval. ^a^ In model 4, we further adjusted for six chronic diseases (diabetes mellitus, hypertension, heart disease, stroke/ cerebrovascular disease, and respiratory diseases) based on model 2 in table 2 (ethnicity, residence, current marital status, education, lifetime primary occupation, economic independence, being in receipt of adequate medication, co-residence with adult children, currently smoking, currently consuming alcohol, BMI, and regular exercise).

^b^ In model 5, we further adjusted for mild cognitive impairment (MCI) based on model 2.

^c^ The cutoff values of serum albumin level were 36.8, 40.1, and 43.4 g/L.

**
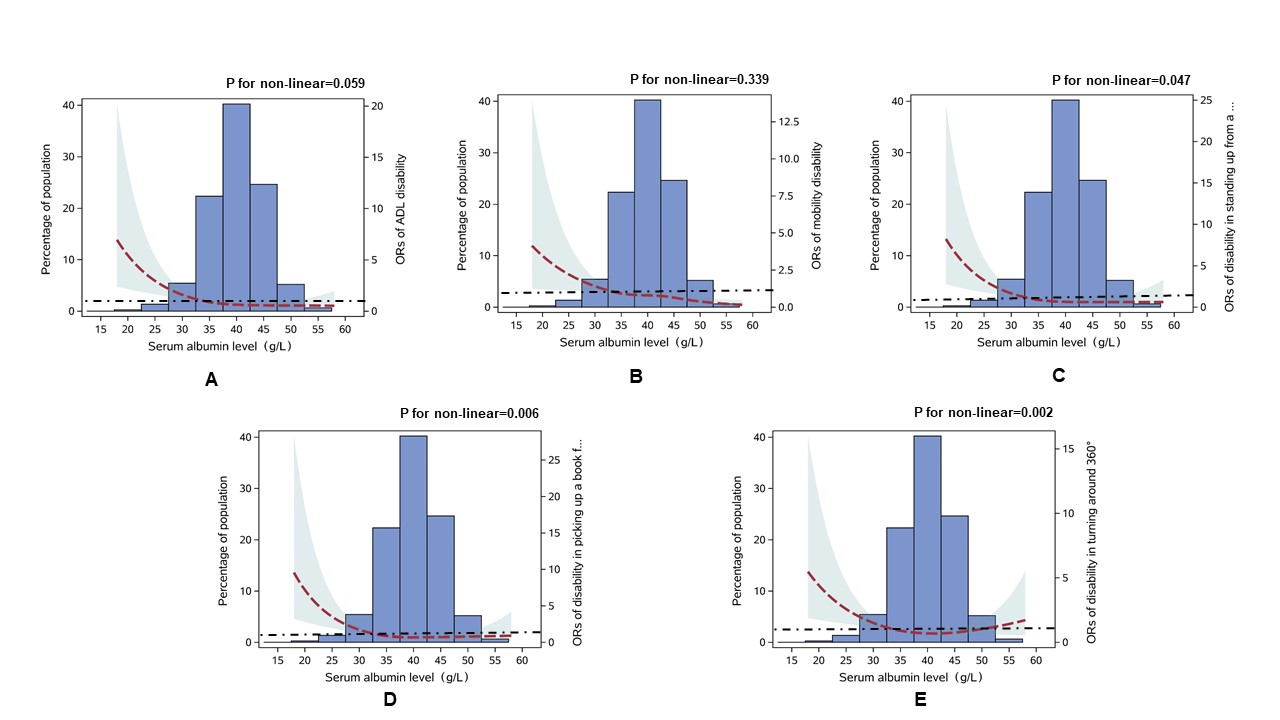
Figure S1. Adjusted dose-response association of serum albumin level with ADL disability, mobility disability, and disability in objective physical functioning.**

A: ADL disability; B: Mobility disability; C: Disability in standing up from a chair; D: Disability in picking up a book from the floor; E: Disability in turning around 360°.

Abbreviations: OR, odds ratio; ADL, activities of daily living.

Note: In model 2, the adjusted dose-response association of serum albumin levels with ADL disability, mobility disability, and disability in objective physical functioning was evaluated. Serum albumin level was coded using a restricted cubic spline (RCS) function with four knots, which approximately corresponded to the P5 (31.2 g/L), P25 (36.8 g/L), P75 (43.3 g/L), and P95 (47.8 g/L) of serum albumin level distribution. The dashed red line represents the adjusted odds ratio of ADL disability, mobility disability, and disability in objective physical functioning for any value of serum albumin level was compared to participants with P10 (33.8 g/L) of serum albumin level. The light blue shaded areas show 95% confidence intervals derived from restricted cubic spline regressions and the blue histograms show the fraction of the population with the different levels of serum albumin. The dashed gray line refers to the reference for the association at an OR of 1.0.
